# DECISION MAKING ABOUT LIFE-SUSTAINING TREATMENT FOR UNREPRESENTED ADULTS IN KOREAN HOSPITALS

**DOI:** 10.1093/geroni/igad104.3013

**Published:** 2023-12-21

**Authors:** Hyejin Kim, Jiyeon Choi, Won Lee, Ilhak Lee, Mi-Kyung Song

**Affiliations:** Chung-Ang University, Seoul, Republic of Korea; Yonsei University, Seoul, Republic of Korea; Chung-Ang University, Seoul, Republic of Korea; Yonsei University, Seoul, Republic of Korea; Emory University, Atlanta, Georgia, United States

## Abstract

**BACKGROUND:**

In South Korea, unrepresented adults are individuals who lack decision-making capacity, written treatment preferences, and available immediate family. Since the enactment of the Life-Sustaining Treatment Decision Act in 2018, ethical and legal dilemmas surrounding decision-making about life-sustaining treatments for unrepresented patients have emerged among healthcare providers in hospitals. Thus, the objectives of this qualitative descriptive study were to explore decision-making practices about life-sustaining treatments for unrepresented patients.

**METHODS:**

We interviewed 37 healthcare providers including physicians, nurses, and social workers from 22 hospitals in South Korea, and analyzed the interview data using a directed content analytic technique. RESULTS: Healthcare providers reported a lack of decision-making guidelines regarding life-sustaining treatments for unrepresented patients in their institutions or being uncertain about the presence of such guidelines. Physicians primarily make decisions about life-sustaining treatments for these patients. Ethics committees’ involvement in such decision-making is minimal in most hospitals. Basic life-sustaining treatments are usually initiated if necessary. However, life-sustaining treatments unrepresented patients are currently receiving cannot be discontinued even though those treatments seem pointless. Thus, additional life-sustaining treatments (e.g., extracorporeal membrane oxygenation) tend to be withheld. Important considerations in the decision-making process include the person’s reversibility, risk of adverse events, and medical costs. CONCLUSION: Decision-making about life-sustaining treatments for unrepresented patients is currently in legally blind spots in South Korea. Reforms in regulations and policies surrounding such decision-making practices for unrepresented patients and the development of institutional guidelines are urgently necessary to improve decision-making practices about life-sustaining treatments for these individuals.

